# Diversity of endophytic fungi and screening of fungal paclitaxel producer from Anglojap yew, *Taxus x media*

**DOI:** 10.1186/1471-2180-13-71

**Published:** 2013-03-28

**Authors:** Zhi-Qiang Xiong, Ying-Ying Yang, Na Zhao, Yong Wang

**Affiliations:** 1 Key Laboratory of Synthetic Biology, Institute of Plant Physiology and Ecology, Shanghai Institutes for Biological Sciences, Chinese Academy of Sciences, Shanghai 200032, China; 2 State Key Laboratory of Bioreactor Engineering, East China University of Science and Technology, Shanghai 200237, China

**Keywords:** *Taxus x media*, Paclitaxel, Endophytic fungi, *Guignardia mangiferae*, Taxol gene cluster

## Abstract

**Background:**

Endophytic fungi represent underexplored resource of novel lead compounds and have a capacity to produce diverse class of plant secondary metabolites. Here we investigated endophytic fungi diversity and screening of paclitaxel-producing fungi from *Taxus x media*.

**Results:**

Eighty-one endophytic fungi isolated from *T. media* were grouped into 8 genera based on the morphological and molecular identification. *Guignardia* and *Colletotrichum* were the dominant genera, whereas the remaining genera were infrequent groups. The genera *Glomerella* and *Gibberella* were first reported in *Taxus*. Three representative species of the distinct genera gave positive hits by molecular marker screening and were capable of producing taxol which were validated by HPLC-MS. Among these 3 taxol-producing fungi, the highest yield of taxol was 720 ng/l by *Guignardia mangiferae* HAA11 compared with those of *Fusarium proliferatum* HBA29 (240 ng/l) and *Colletotrichum gloeosporioides* TA67 (120 ng/l). This is the first report of taxol producer from *Guignardia*. Moreover, the lower similarities of *ts* and *bapt* between microbial and plant origin suggested that fungal taxol biosynthetic cluster might be repeatedly invented during evolution, nor horizontal gene transfer from *Taxus* species.

**Conclusions:**

Taxol-producing endophytic fungi could be a fascinating reservoir to generate taxol-related drug lead and to elucidate the remained 5 unknown genes or the potential regulation mechanism in the taxol biosynthesis pathway.

## Background

Plant-associated microorganisms, especially endophytic fungi, are largely underexplored in the discovery of natural products [1]. The prolific endophytes also have a capacity to produce diverse class of plant associated secondary metabolites with a wide variety of biological activities such as antimicrobial agent hypericin [2], acetylcholinesterase inhibitor huperzine A [3], and antitumor agents taxol [4]. Bioprospecting endophytes thus offers tremendous promise to discover natural products with therapeutic value [1], which have attracted increasing attention among microbiologists, ecologists, agronomists, and chemists.

Among plant-derived natural products, taxol (a blockbuster anticancer drug) is widely used for clinical application against different types of cancer [5]. It was originally obtained from extraction of the bark of *Taxus* species. However, mass production of taxol remains a vexing problem due to low taxol content in the *Taxus* species. 13,500 kg of *T. brevifolia* (Pacific yew, the most productive species) bark only yields about 1 kg of taxol [6], whereas at least 2 g of taxol is required for a full regimen of antitumor treatment in a patient [4]. With the increasing demand for taxol and the shortage of plant resource, there is an urgent need to find other alternative production methods.

Several alternative strategies have been developed for taxol production during the past two decades. Total chemical synthesis is available [7], but the large number of reaction steps and low yield limit its practicality. Semisynthesis from taxol precursors baccatin III or 10-deacetylbaccatin III solves the supply problem of taxol which appears so formidable, but still relies on plant precursor compounds with difficulty in the purification process [8]. Plant tissue culture as an environmentally sustainable method is successfully developed for large-scale taxol production, but long incubation time and low yield render it an economic impossibility [9]. Notwithstanding the remarkable progress in the different production alternatives, these methods are not enabled to meet the increasing taxol demand with an economic supply [10]. Consequently, more production options are still required to lower the price of taxol and increase its availability.

*Taxomyces andreanae* is the first report of a microbial taxol producer from Pacific yew [4], implying that microorganisms as a potential source would be one of the most desirable means for taxol supply. Potential advantages of microbial taxol production include a fast growth at high cell density cultivation, easy genetic manipulation, and the possibility of scale-up on an industrial level [10]. In addition, microbial production helps to protect natural plant *Taxus* resources [11]. Current research in this field is focused on screening taxol-producing endophytic microbes [4], improving taxol yield by genome shuffling [12], genetic engineering [13], and process optimization [14], and heterologous expression of taxol precursor in microorganisms [15].

**Figure 1:**
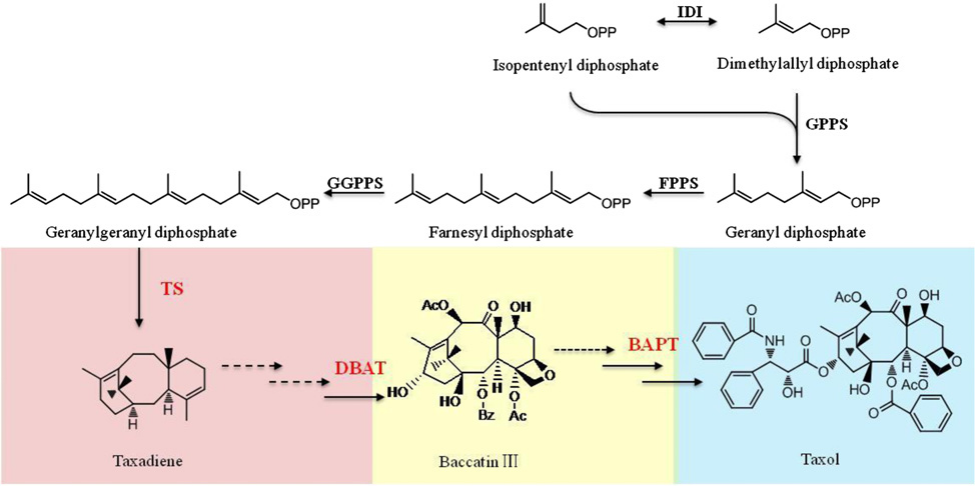
Key genes in the taxol biosynthetic pathway.

Isolation of endophytic microorganisms is a comparatively simple process, but taxol detection of all isolates is laborious [16]. Compared to this traditional screening method, the molecular marker screening is an efficient alternative method to find taxol-producing microbes [17]. Three probes based on key genes of taxol biosynthetic cluster, *ts* (encoding taxadiene synthase), *dbat* (encoding 10-deacetylbaccatin III-10-O-acetyltransferase), and *bapt* (encoding C-13 phenylpropanoyl side chain-CoA acyltransferase), have been applied in the primary screening of taxol-producing endophytic microorganisms (Figure 1). Currently, more than 30 endophytic microorganisms have been reported to produce taxol with ranging from 10 ng/l to 800 μg/l through the traditional screening/molecular marker screening [10]; the majority of endophytic microbes belong to fungi such as ascomycetes and imperfect fungi, and a minority are bacteria such as *Kitasatospora* and *Streptomyces*. However, to date, there are only a few reports to investigate biodiversity of microorganisms living in *Taxus*[18]. In this work, we surveyed the endophytic fungi diversity of *T. media*, and discovered taxol-producing endophytes from the fungal isolates based on molecular markers derived from key biosynthetic enzymes of taxol. To our knowledge, *Guignardia* is the first report to produce taxol.

## Results and discussion

### Endophytic fungal diversity of *T. media*

**Figure 2:**
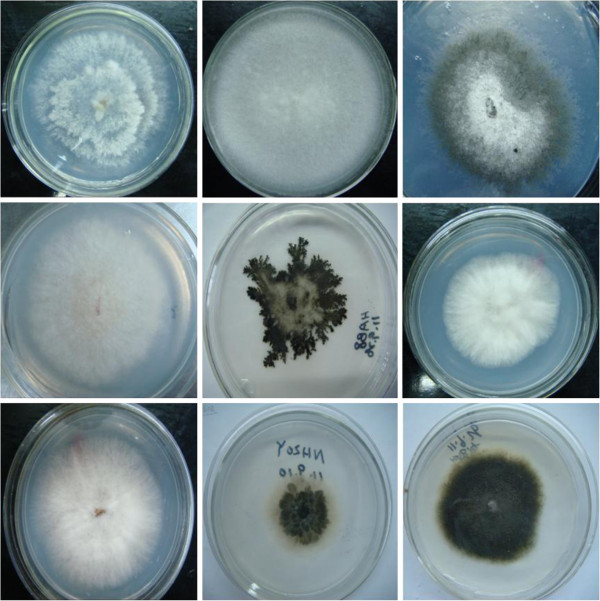
**Morphological characteristics of fungal endophytes in *T. media*.**

**Figure 3:**
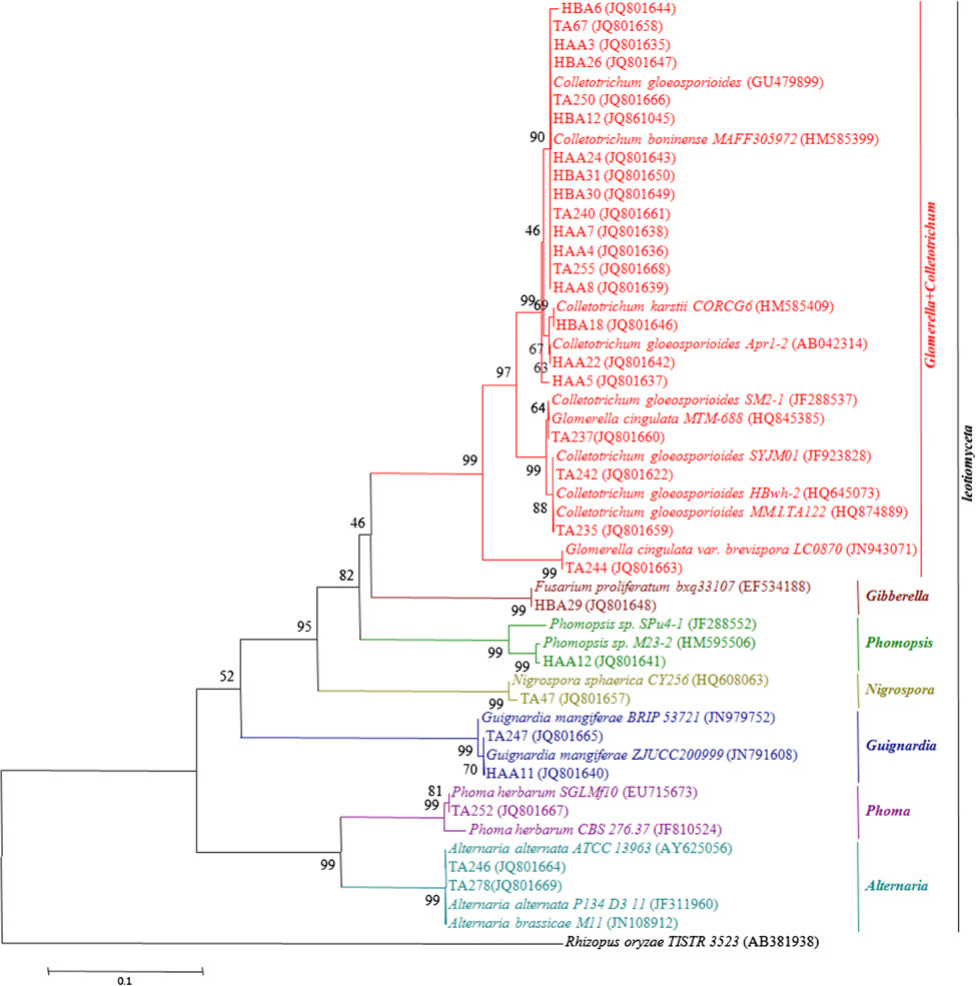
Molecular identification of the 29 morphotypes based on ITS rDNA sequence analysis.

**Figure 4:**
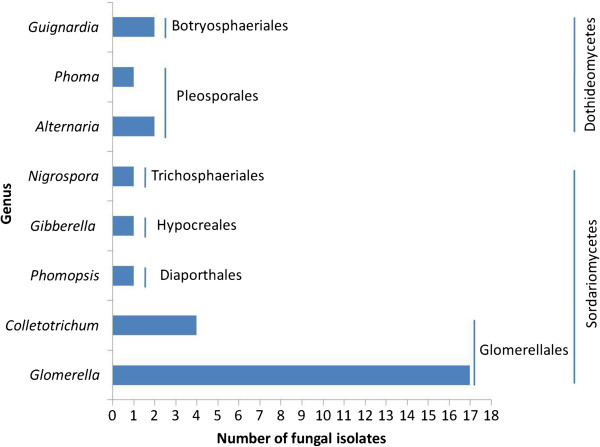
The frequency of ITS-based genotypes determined from the 29 morphotypes.

To assess the presence of fungal endophytes in *T. media*, 81 fungal isolates were recovered and assigned to 29 morphotypes using dereplication based on the morphological characteristics and unique phenotypic characters (Figure 2). The identified fungi belonged to the phylum Ascomycota. To confirm the reliability of morphological identification, all 29 morphotypes (strains HAA3, HAA4, HAA5, HAA7, HAA8, HAA11, HAA12, HAA22, HAA24, HBA6, HBA12, HBA18, HBA26, HBA29, HBA30, HBA31, TA47, TA67, TA235, TA237, TA240, TA242, TA244, TA246, TA247, TA250, TA252, TA255, and TA278) were subjected to molecular identification based on ITS rDNA sequence analysis (Figure 3). The 29 morphospecies were grouped into 8 genera (*Alternaria*, *Colletotrichum*, *Glomerella*, *Gibberella*, *Guignardia*, *Nigrospora*, *Phomopsis*, and *Phoma*). Analysis of distribution frequencies of the 29 morphotypes revealed that the fungal communities in the host contained two frequent genera and many infrequent groups (Figure 4). *Glomerella* and *Colletotrichum* were the dominant genera, accounting for 13.8% and 58.6% of colonization frequencies (Table 1). Among the rare genera, *Alternaria* and *Guignardia* represented ~6.9% of isolation frequencies, whereas others showed ~3.4% of colonization frequencies (Table 1). Our result confirmed that a few species are frequent colonizers, and yet the majority are rare inhabitants in woody plants [18].

**Table 1:** Putative taxonomic affinities and frequency of the 29 morphotypes

**Fungal isolate**	**Accession number**	**Closest relatives in NCBI**	**ITS identity (%)**	**Frequency**	**Genus**
HAA3	JQ801635	*Colletotrichum boninense* MAFF305972 (HM585399)	100%	34.5%	*Colletotrichum*
HAA4	JQ801636	*Colletotrichum boninense* MAFF305972 (HM585399)	100%		
HAA7	JQ801638	*Colletotrichum boninense* MAFF305972 (HM585399)	100%		
HAA8	JQ801639	*Colletotrichum boninense* MAFF305972 (HM585399)	100%		
HAA24	JQ801643	*Colletotrichum boninense* MAFF305972 (HM585399)	100%		
HBA12	JQ801645	*Colletotrichum boninense* MAFF305972 (HM585399)	100%		
HBA26	JQ801647	*Colletotrichum boninense* MAFF305972 (HM585399)	100%		
HBA30	JQ801649	*Colletotrichum boninense* MAFF305972 (HM585399)	100%		
HBA31	JQ801650	*Colletotrichum boninense* MAFF305972 (HM585399)	100%		
HBA6	JQ801644	*Colletotrichum boninense* MAFF305972 (HM585399)	99%		
HAA5	JQ801637	*Colletotrichum petchii* CBS:378.94 (JQ005223)	99%	3.4%	
HBA18	JQ801646	*Colletorichum karstii* CORCG6 (HM585409)	100%	3.4%	
TA67	JQ801658	*Colletotrichum gloeosporioides* (GU479899)	100%	17.2%	
TA240	JQ801661	*Colletotrichum gloeosporioides* (GU479899)	99%		
TA250	JQ801666	*Colletotrichum gloeosporioides* (GU479899)	100%		
TA255	JQ801668	*Colletotrichum gloeosporioides* (GU479899)	99%		
TA242	JQ801662	*Colletotrichum gloeosporioides* MM.I.TA122 (HQ874889)	100%		
HAA11	JQ801640	*Guignardia mangiferae* ZJUCC200999 (JN791608)	100%	6.9%	*Guignardia*
TA247	JQ801665	*Guignardia mangiferae* ZJUCC200999 (JN791608)	100%		
HAA12	JQ801641	*Phomopsis* sp. M23-2 (HM595506)	99%	3.4%	*Phomopsis*
HAA22	JQ801642	*Glomerella* sp. HS-EF2 (GQ334409)	100%	3.4%	*Glomerella*
TA237	JQ801660	*Glomerella cingulata* MTM-688 (HQ845385)	100%	10.3%	
TA235	JQ801659	*Glomerella cingulata* MAFF 305913 (AB042315)	99%		
TA244	JQ801663	*Glomerella cingulata* var. *brevispora* LC0870 (JN943071)	100%		
HBA29	JQ801648	*Fusarium proliferatum* bxq33107 (EF534188)	100%	3.4%	*Gibberella*
TA47	JQ801657	*Nigrospora sphaerica* CY256 (HQ608063)	99%	3.4%	*Nigrospora*
TA246	JQ801664	*Alternaria brassicae* M11 (JN108912)	100%	3.4%	*Alternaria*
TA278	JQ801669	*Alternaria alternata* P143_D3_11 (JF311960)	100%	3.4%	
TA252	JQ801667	*Phoma herbarum* SGLMf10 (EU715673)	99%	3.4%	*Phoma*

Although *Glomerella* and *Colletotrichum* are frequent colonizers in *T. media* (temperate regions) in this study, they are not cosmopolitan species within other *Taxus* plants [18,19], such as the frequent genera *Diaporthe*, *Phomopsis*, *Acremonium*, and *Pezicula* in *T. chinensis* (mountain region of Qinba, northern-western China), and *Myceliasterilia*, *Alternaria*, and *Fusarium* in *T. baccata* and *T. brevifolia* (central-northern Italy), indicating that the dominant genera are distinct in different yews and different geographic region [20]. The genera *Glomerella* and *Gibberella* were first reported endophytes in *Taxus*, but they have been isolated from other host plants [21,22].

In total, 11 distinctive genotypes were detected at a 99% sequence similarity threshold (Figure 3), which did not correspond well with morphological differences between these fungal cultures. Strains HAA12, HBA29, TA47, TA244, TA246, and TA278 were located with a high bootstrap support (99-100%) in their own cluster, while strains HAA11, HAA22, HBA18, TA67, TA235, TA237, TA240, TA242, TA250, and TA255 formed their own cluster with a bootstrap value from 70 to 99%. Strains HAA3, HAA4, HAA5, HAA7, HAA8, HAA24, HBA6, HBA12, HBA26, HBA30, and HBA31 were clustered to *Colletotrichum boninense* with a bootstrap value of 90%, but sequence identities with the available references in NCBI were very high (100%). In addition, strain HAA5 was not similar to any references with a bootstrap value of 63%, but shared sequence similarities of 99% with *C. boninens*. It might represent novel species or even new genera.

### Primary screening of taxol-producing fungi based on molecular marker

Molecular marker based screening is a rapid and efficient alternative to find taxol-producing endophytic microbes in contrast to the traditional screening method [11,17]. This method is not dependent on the production of paclitaxel and can indicate the presence of some required genes for taxol biosynthesis in the microbial genome. In yew trees, taxol biosynthesis involves 19 enzymatic steps from the universal diterpenoid precursor geranylgeranyl diphosphate (GGPP) by the plastidial methyl erythritol phosphate pathway [23]. We thus chose *ts* (involved in formation of the taxane skeleton), *dbat* (involved in baccatin III formation), and *bapt* (involved in phenylpropanoyl side chain formation at C13), three key genes in taxol biosynthesis, as a primary screening to identify taxol-producing fungi.

**Figure 5:**
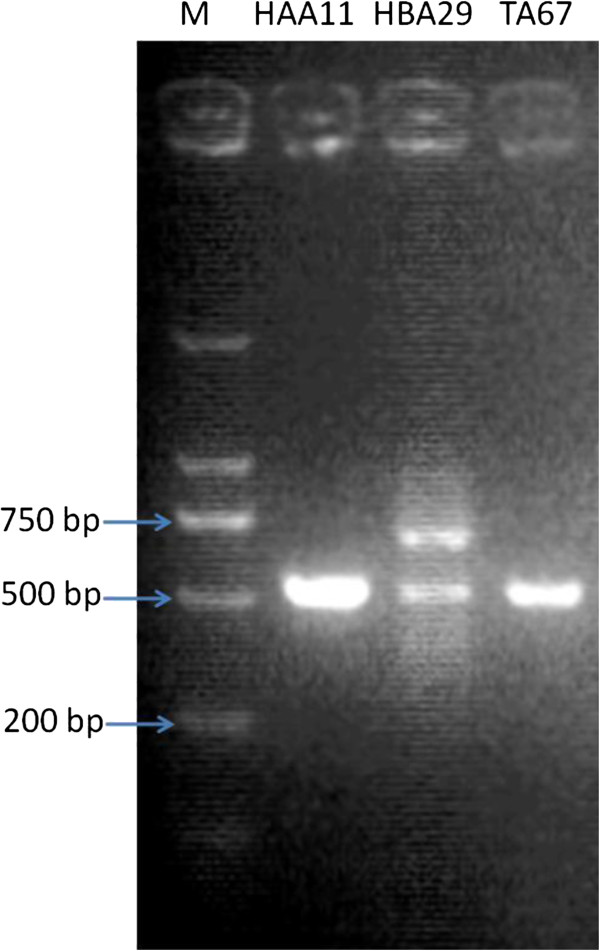
**PCR analysis for the presence of *bapt *in endophytic fungi from *T. media*.** Ladder M: DS2000 DNA marker (Dongsheng Biotech Ltd, China); Lane 1–3, the PCR product of strains HAA11, HBA29, and TA67.

All 11 fungal isolates with distinctive genotype separated from *T. media* were consecutively screened for the presence of *ts*, *dbat*, and *bapt* genes. Three fungi (strains HAA11, HBA29, and TA67) had positive hits of *ts* and *dbat*. The *ts* and *dbat* genes are essential for taxol biosynthesis but not diagnostic because taxol precursor baccatin III producers also have *ts* and *dbat*. Thus, the 3 fungi were screened for the presence of *bapt*. Interestingly, all these 3 fungi had approximately 530 bp fragments of *bapt* gene (Figure 5), suggesting that all of them may produce taxol. Currently, only *ts*, *dbat*, and *bapt* genes have been used as molecular probes for the primary screening of taxol producing microorganisms [16,17], thus designing suitable degenerate primers for amplification of more target genes, e.g., the final acylation step in taxol biosynthesis, taxoid C13-side-chain N-benzoyltransferase (DBTNBT), may be a better option for screening.

### Identification of fungal taxol

**Figure 6:**
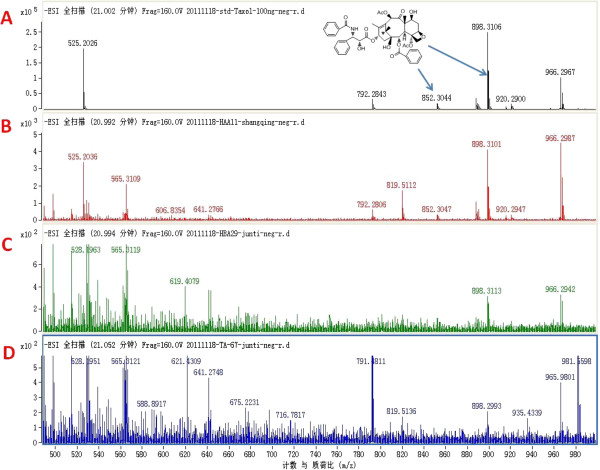
**Mass spectrometric analysis of authentic taxol (A) and the fungal isolates sample solution of HAA11 (B), HBA29 (C), and TA67 (D).** The arrows indicate the identical peak of mass spectroscopy of taxol.

We screened the extracts of the 3 representative species *Guignardia mangiferae* HAA11, *Fusarium proliferatum* HBA29, and *Colletotrichum gloeosporioides* TA67 with positive results in the primary screening to detect fungal taxol by high performance liquid chromatography-mass spectrometry (LC-MS). The HPLC peak positions and peak shapes of the 3 representative species from the different genera were identical to that of standard taxol (retention time = 21.02±0.03 min), indicating the 3 distinct fungi may produce taxol. Further convincing evidence for the identity of the fungal taxol was obtained by high resolution MS (Figure 6). Characteristically, the authentic taxol yielded an [M-H]^-^ peak at *m/z* 852.32 and an [M+COOH]^-^ peak at *m/z* 898.32. By comparison, the fungal taxol also yielded a peak at *m/z* 852.32 ± 0.03 and a characteristic fragment peak at *m/z* 898.32 ± 0.02 (Table 2). The peaks of fungal taxol exhibited *m/z* ratios corresponding to the molecular ions of standard taxol, demonstrating that the 3 fungal endophytes can generate taxol in vitro. Among these 3 taxol-producing fungi, strain HAA11 had the highest taxol yield (720 ng/l) in the PDB medium in comparison with those of strains HBA29 (240 ng/l) and TA67 (120 ng/l).

**Table 2:** The mass spectral fragment ions of taxol

**Fragment peak**							
**Standard**		**HAA-11**		**HBA-29**		**TA-67**	
(M-H)^-^	(M+COOH)^-^	(M-H)^-^	(M+COOH)^-^	(M-H)^-^	(M+COOH)^-^	(M-H)^-^	(M+COOH)^-^
852.32	898.32	852.29	898.30	-	898.30	-	898.31

*Colletotrichum gloeosporioides* has been proven to be capable of producing taxol (163.4 μg/l) [24]. *Guignardia mangiferae* and *Fusarium proliferatum* have not been obtained from other yews and some reported taxol-producing fungi from other *Taxus* plants have not been isolated from *T. media* in this work, suggesting that yews in different geographic regions can harbor novel and highly diverse taxol producing fungi and certain taxol-generating fungi may be host-specific. Thus, to isolate taxol-producing fungal species, more consideration should be given to different hosts under different conditions. In addition, *Guignardia mangiferae* HAA11 and *Fusarium proliferatum* HBA29 were recovered as infrequent genera, indicating that infrequent genera from *Taxus* might be a huge source of taxol-producing fungi [18].

Although taxol concentration of *Guignardia mangiferae* HAA11, *Fusarium proliferatum* HBA29, and *Colletotrichum gloeosporioides* TA67 is relatively lower than that of *Taxus* species, the high growth rate and short generation time make them worthwhile to continue investigation. Thus, to meet the commercial need for taxol, further work will focus on improving taxol yield in fungi by combination of various biotechnological approaches such as strain improvement, genetic manipulation, and fermentation engineering. In addition, the lack of a complete taxol biosynthetic cluster (5 unknown enzymatic steps) is at present a bottleneck for basic and applied research, genome sequencing and analysis of taxol-producing microorganisms (the relatively small genomes) thus could significantly expand the number of known taxol biosynthetic genes to elucidate the whole pathway and provide the basis for heterologous production. From an evolutionary adaptation point of view, endophytic microorganism harboring particular metabolic functions could be achieved extraordinary success in occupying a niche within plant tissues or even contribute to host defense against various invading pathogens [18], hence, analysis of genes involved in taxol synthesis from the diverse fungi will significantly enhance our understanding of the co-evolutionary mechanism of the endophyte host [11].

### *ts* and *bapt* genes between taxol-producing fungi and *Taxus*

The amplified DNA fragments of *ts* (from strain HBA29) and *bapt* (from strains HAA11 and TA67) were sequenced and analyzed using Blastn in the NCBI database. The *ts* segment from strain HBA29 shares 40.6% identity with cDNA of *ts* from *T. media* [GenBank accession no. AY461450]. The *bapt* segments from strains HAA11 and TA67 have lower identity (40.0% and 44.1%, respectively) with cDNA of *bapt* from *T. media* [GenBank accession no. AY563630], indicating that it might be a fragment of the new putative fungal *bapt* gene. Despite our findings are contrary to all previous works of *ts* and *bapt* from endophytic fungi which show high homology (> 96% sequence identity) with theirs plant counterparts [10,16,25-27], the success of our screening for microbial *ts*, *dbat* and *bapt* using the designed PCR primer based on the conserved regions of key genes of taxol biosynthetic pathway in yew provides crucial evidence for the molecular blueprint of taxol biosynthesis being an inherent genetic trait of endophytic fungi. Moreover, the detection of taxol production affords definitive proof for the presence of taxol pathway in endophytic fungi. Consequently, low similarity of *ts* and *bapt* between plant and microbial origin seems to give a new insight to the controversial hypothesis of horizontal gene transfer (HGT). The evolutionary trajectory of taxol gene cluster between microbial and plant origin might be coexisting. Although HGT in fungi are largely reported [28], the ultimate plausibility of microbial taxol gene cluster by HGT hypothesis should be revisited and further investigated because approximately 20 genes involved in the taxol biosynthesis make HGT rather unlikely. Additionally, taxol-producing endophytic fungi have been isolated from plants which themselves are not capable of producing taxol [29-34], suggesting that taxol biosynthesis in fungi may not be acquired from HGT. In nature, gibberellin biosynthetic pathways in fungi and higher plants have evolved independently and not by HGT [35,36]. We thus assumed that taxol biosynthetic cluster might be repeatedly invented during evolution. Moreover, it raises an intriguing question: whether the genes responsible for fungal taxol biosynthesis are indeed grouped in a contiguous cluster?

## Conclusions

Eighty-one endophytic fungi isolated from *T. media* were grouped into 8 genera based on the morphological and molecular identification. *Guignardia* and *Colletotrichum* were the dominant genera, whereas the remaining genera were infrequent groups. Three representative species of the distinct genera can produce taxol. This is the first report of taxol prodcer from *Guignardia*. The highest taxol yield was 720 ng/l by *Guignardia mangiferae* HAA-11. Moreover, the lower similarities of *ts* and *bapt* between microbial and plant origin indicated that fungal taxol biosynthetic cluster might be repeatedly invented during evolution, nor HGT from *Taxus* species.

## Methods

### Isolation of endophytic fungi from *T. media*

Plant samples including the bark pieces and leaves were collected from *T. media* (Shanghai, China). The samples were treated with 75% ethanol (v/v) for 1 min and 2.5% sodium hypochlorite (v/v) for 2 min, and rinsed two times in sterilized water. In order to test the effectiveness of surface sterilization [21], sterilized samples were imprinted onto potato dextrose agar with 100 μg/l streptomycin (PDAS) in Petri dishes at 28°C for 1 week. In addition, 10 ml of the last rinsing water were centrifuged for 10 min at 5000 rpm. The supernatant was removed and added 500 μl sterilized water in the centrifugal tube; 100 μl of this volume were then plated onto PDAS. The surface sterilization was validated because no mycelial growth occurred. The surface-disinfected small pieces (4 mm^2^) of inner bark and leaf segments were excised and placed on the surface of PDAS in Petri dishes, incubated at 28°C for 3–7 days to allow the growth of endophytic fungi, and periodically checked for culture purity. Pure fungal cultures of the endophytic isolates were obtained by the hyphal tip method [37]. All fungal isolates were numbered and stored in 15% (v/v) glycerol at −80°C as spores and mycelium.

### Identification of endophytic fungi from *T. media*

Individual hyphal tips of various fungal isolates were subcultured onto fresh PDA medium, and incubated at 28°C for at least 2 weeks. All fungal isolates were initially identified to the genus and/or species level based on morphology of fungal colony, characteristics of fungal spore, and molecular phylogenetic analysis. The fungal isolates were inoculated individually into 250 ml Erlenmeyer flasks containing 25 ml potato dextrose broth (PDB) medium. Cultures were incubated at 200 rpm at 28°C for 2 days and harvested by centrifugation at 12000 r/min for 10 min.

Genomic DNA was extracted from 0.5-1 g chilled mycelia in liquid nitrogen using the SDS-CTAB method [38]. The fungal internal transcribed spacer (ITS) fragments (ITS1-5.8S-ITS2 rDNA) were amplified by PCR using the universal primers ITS1 and ITS4 (Table 3). The PCR reaction mixtures (25 μl) consisted of 1 μl genomic DNA (~100 ng), 0.5 μl forward and reverse primers (20 μM), and 12.5 μl *Premix Taq* (TaKaRa Biotechnology Ltd., China), and 10.5 μl PCR quality water. The PCR reaction programs were pre-heating at 94°C for 3 min, 30 cycles of 94°C for 30 s, 55°C for 30 s, 72°C for 1 min, and a final extension at 72°C for 5 min. The PCR products were analyzed by agarose gel electrophoresis and purified using a DNA gel exaction kit (Axygen Biotechnology Ltd., China). The purified PCR product was directly sequenced using the same primers by BGI-Shanghai (Shanghai, China).

**Table 3:** Oligonucleotide primers used in PCR screening

**Gene (GenBank No.)**	**Primers**	**Sequence (5′-3′)**	**Amplicon length**
ITS1-5.8S-ITS2 rDNA	ITS1	TCCGTAGGTGAACCTGCGG	500-600 bp [39]
	ITS4	TCCTCCGCTTATTGATATGC	
*ts* (AY364469)	ts-F	CAAACCCATGTCGAATTGAGAAG	631 bp [16]
	ts-R	CAAGTTTGCATACACTCTGGAATCT	
*dbat* (EF028093)	dbat-F	GGGAGGGTGCTCTGTTTG	153 bp [17]
	dbat-R	GTTACCTGAACCACCAGAGG	
*bapt* (AY082804)	bapt-F	CCTCTCTCCGCCATTGACAA	453 bp [17]
	bapt-R	TCGCCATCTCTGCCATACTT	

The ITS sequences of endophytic fungi were compared with the data in National Center for Biotechnology Information, USA (NCBI) using BLAST search (http://blast.ncbi.nlm.nih.gov/Blast.cgi) to estimate the phylogenetic relationship. CLUSTAL X software (version 2.0, Conway Institute, USA) was used to generate alignment of endophytic fungi [40]. Phylogenetic analysis was carried out by the neighbor-joining method using MEGA software (version 4.0, Biodesign Institute, USA). The bootstrap was 1,000 replications to assess the reliable level to the nods of the tree [41].

### Primary screening of taxol-producing fungi based on PCR amplification

The conserved sequences of three key genes in the taxol biosynthetic pathway, *ts*, *dbat*, and *bapt*, were used as molecular markers to PCR amplification for primary screening of taxol-producing fungi. The specific primers ts-F, ts-R, dbat-F, dbat-R, bapt-F, bapt-R (Table 3) were synthesized by Sangon Biotech Co., Ltd. (Shanghai, China). PCR amplification was performed in a Mastercycler personal Thermal Cycler (Eppendorf Inc., Germany).The fungal isolates were firstly screened for the presence of *ts* gene, secondly screened for *bapt* gene, and lastly screened for *dbat* gene. PCR amplification was carried out according to previously reported PCR conditions in the literatures [16,17]. PCR products were analyzed on 2% (wt/vol) agarose gel and purified by DNA gel exaction kit (Axygen). The purified PCR products were ligated to pMD19-T vectors (TaKaRa), transformed into *E. coli* DH10B, and sequenced by BGI-Shanghai. Those fungi with PCR positive for molecular makers were selected for the next screening.

### Determination of Taxol-producing fungi

Three fungi with positive results of primary screening were inoculated into 250 ml Erlenmeyer flasks containing 25ml PDB medium to detect taxol production. The culture condition of fungal endophytes was the same as mentioned above, except that the culture time was changed to 5 days. The mycelia were harvested by centrifugation and freezed by liquid nitrogen, then thoroughly crushed in a mortar. The fermentation broths and ground mycelia were extracted with ethyl acetate 3 times at room temperature. All extracts were combined and concentrated under reduced pressure, and redissolved with 0.5 ml of 100% methanol (v/v).

The extracts of each fungal isolate were examined for the presence of taxol using HPLC-MS. A C_18_ column (4.6×50 mm, 1.8μm particle size, Zorbax XDB, Agilent) was used to identify taxol by HPLC [11]. The methanol solution of putative taxol (5 μl) were injected and elution was done with methanol/H_2_O binary solvent-delivery gradient elution (0–20 min, 5%-100% methanol; 20–25 min, 100% methanol; 25–35 min, 5% methanol; volume fraction). The wavelength at 226 nm was used to detect compounds eluting from the column. Electrospray mass spectroscopy was done on fungal taxol samples using the electrospray technique with an Agilent 1100 LC/MSD trap. The sample in 100% methanol was injected with a spray flow of 2 μl/min and a spray voltage of 2.2 kV by the loop injection method. The mass spectral fragment ions of taxol are shown in Table 2.

### Nucleotide sequence accession numbers

The partial sequences of the ITS rDNA, *ts*, and *bapt* genes obtained from cultures and clones were deposited in GenBank (NCBI) under the accession numbers JQ801635-JQ801669 and KC337343-KC337345.

## Competing interest

The authors declare that they have no competing interest.

## Authors’ contributions

ZQX collected plant samples and designed the experiments; YYY isolated and characterized of endophytic fungi. NZ performed fungal cultivation. ZQX and YW were involved in conception and interpretation of the results and drafting the manuscript. ZQX and YW were involved in critically revision the manuscript and approved the manuscript for publication. All authors read and approved the final manuscript.
